# Therapeutic Granulomonocytapheresis as a Non-pharmacologic Treatment Option for Inflammatory Bowel Disease: Efficacy Reports on a Wide Age Range and Disease Profile

**DOI:** 10.7759/cureus.48913

**Published:** 2023-11-16

**Authors:** Tomotaka Tanaka

**Affiliations:** 1 Department of Gastroenterology, Tsuchiya General Hospital, Hiroshima, JPN

**Keywords:** myeloid lineage leukocytes, regulatory b-cells, pediatrics and adolescents, mucosal lesions, interleukin-10, apoptotic neutrophils, therapeutic granulomonocytapheresis, inflammatory bowel disease

## Abstract

The major phenotypes of inflammatory bowel disease (IBD) include ulcerative colitis (UC) and Crohn’s disease (CD), which cause debilitating symptoms, including bloody diarrhea, abdominal discomfort, and fever. Patients require life-long immunosuppressive medications, which cause adverse side effects as additional morbidity factors. However, IBD is initiated and perpetuated by inflammatory cytokines, and given that in patients with IBD myeloid lineage leukocytes are elevated with activation behavior and release inflammatory cytokines, selective depletion of elevated granulocytes and monocytes by granulomonocytapheresis is a relevant therapeutic option for IBD patients.

Therefore, a column filled with specially designed beads as granulomonocytapheresis carriers for selective adsorption of myeloid lineage leukocytes (Adacolumn) has been applied to treat patients with active IBD. Patients receive up to 10 granulomonocytapheresis sessions at one or two sessions per week. During each session, the carriers adsorb up to 60% of the myeloid leukocytes from the blood that passes through the granulomonocytapheresis column.

Efficacy rates in the UC setting have been as high as 85% in steroid-naïve patients, and 100% in drug-naïve, first-episode cases, but patients with a long duration of active IBD and extensive colonic lesions that have become refractory to pharmacological treatment have not responded well. However, granulomonocytapheresis has a favorable safety profile.

Given that immunosuppressive medications used to treat IBD potentially may increase the risk of severe viral infection, non-drug granulomonocytapheresis should be a favorable treatment strategy. Further, by targeting granulomonocytapheresis to patients with background features and identifying a patient as a likely responder, futile use of medical resources is avoided.

## Introduction and background

The major phenotypes of inflammatory bowel disease (IBD) are ulcerative colitis (UC) and Crohn’s disease (CD), which afflict millions of individuals throughout the world with debilitating symptoms [1-3]. Further, although the expression of UC is confined to the large intestine, CD may appear in any part of the digestive system, from the mouth to the perianal region, and more than 65% of the patients may have small intestinal involvement [2].

**Figure 1:**
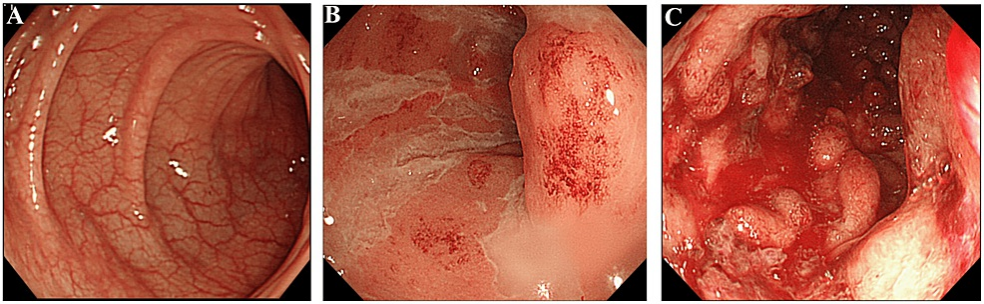
Colonoscopy images Colonoscopy images showing visible mucosal vascular patterns in an individual during complete remission (A). The blood vessels ensure optimum absorption of water and nutrients from the intestine. Accordingly, the intestinal mucosa in healthy individuals is typically well vascularized for adequate absorption. In ulcerative colitis patients, inflammation in the intestinal mucosa is followed by loss of visible vascular patterns (B). The inflammation, if untreated, leads to bleeding ulcers and loss of the mucosal tissue (C). The images are taken by the author and used with the permission of the patient.

**Figure 2:**
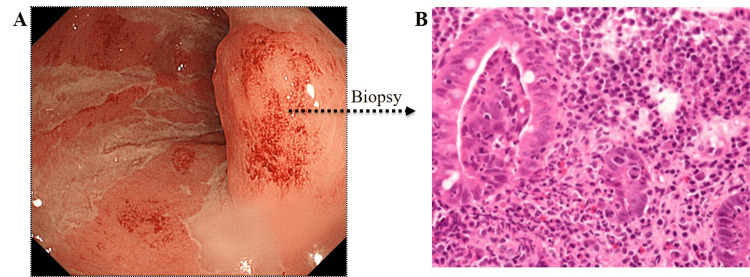
Mucosal tissue biopsy A biopsy of the mucosal tissue from the colonoscopy image in Figure 1B showing a high density of infiltrated myeloid lineage leukocytes. The infiltrated leukocytes are overwhelmingly neutrophils and monocytes/macrophages. The images are taken by the author and used with the permission of the patient.

However, the prevalence of IBD throughout the world is increasing, reflecting the emergence of IBD as a major morbidity [3]. In Figure 1, for comparison, colonoscopy images from an IBD patient during complete remission (Figure 1A) together with images from patients with active UC (Figures 1B, 1C) are presented, while in Figure 2, a biopsy specimen shows infiltration of the intestinal mucosa by myeloid lineage leukocytes, contributing to inflammation and tissue injury. Normal mucosa is highly vascularized for optimum absorption of water and nutrients from the intestine into the bloodstream. In contrast, the intestinal mucosa in patients with IBD during active disease shows inflammation and loss of the visible vascular patterns, which, if untreated, leads to loss of the mucosal tissue, as seen in Figure 1C. Both mucosal inflammation and ulcers lead to inadequate absorption of water and nutrients from the gut. The affected patients may lose weight and become anemic due to bloody diarrhea, a debilitating condition.

## Review

The prevalence of IBD in pediatrics and adolescents

Epidemiological studies show that up to 30% of CD and 20% of UC patients are younger than 20 years of age [4]. Further, over the past decade, data from pediatric IBD studies have revealed differences in the genetic profiles, clinicopathologic features, and natural history between pediatric and adult IBD patients [5,6]. Factors associated with these differences remain unexplained at present. Nonetheless, the location of pediatric UC may differ from that of adult UC [5-7]. Pediatric UC more often appears as pancolitis (up to 90% of patients), while left-sided colitis and proctitis are more common in adults. In light of these realities, clinical investigators continue focusing on pediatric IBD because a better understanding is expected to shed light on the predisposing factors, future disease behavior, and how best to treat IBD [5]. Additionally, growth failure, delayed puberty, and impaired mental development are reported in pediatrics with IBD, which may be reversed if the disease is controlled, the patient receives nutritional support, and is spared from exposure to glucocorticoids [6-8].

In this article, we have briefly reviewed the clinical presentation and treatment options for IBD in the pediatric and adolescent settings and then focused on the potential of granulomonocytapheresis with Adacolumn as a non-pharmacological treatment option for growing patients [9]. Given its emergence as an effective therapeutic option with an unrivaled safety profile in clinical practice, granulomonocytapheresis holds the promise of treatment without drugs [10-15].

However, with regard to drug therapy, despite differences between pediatric and adult IBD, treatment has been based on the same paradigms as for adults with IBD [15-22]. According to the published guidelines [23-25], in the pediatric UC setting with mild to moderate distal colitis, oral or topical 5-aminosalicylates (5-ASA) preparations as suppositories for proctitis, and enema for the left-sided colitis are appropriate first-line remission induction interventions [23]. In fact, for severe pancolitis, corticosteroids have been the first-line medication. When the response is inadequate, other options include tacrolimus, methotrexate, intravenous cyclosporine A, and anti-tumor necrosis factor (anti-TNF)-α biologics [23,24]. However, it is essential to bear in mind that the treatment of pediatric patients with immunomodulators or biologics may carry an increased risk of bacterial, fungal, or viral infection, in addition to adversely impacting the patient’s growth and development [26-28].

The logic of granulomonocytapheresis for IBD

The chronic relapsing-remitting nature of IBD reflects a dysregulated immune response to host gut microbiota together with other genetic and environmental predisposing factors [2,29-31]. Accordingly, patients with IBD bear an overactive immune profile, in particular compromised lymphocyte count [9,15,32-34], while granulocytes and monocytes are elevated (Table 1), showing activation behavior and increased survival time [15,21]. Additionally, there are sub-populations of lymphocytes like the CD4(+)CD25(+) phenotype known as the regulatory T-cells (Treg), as well as B-cells (Breg), which have essential immunoregulatory effects [35-37]. With these in mind, granulomonocytapheresis is designed to spare lymphocytes and boost the immunoregulatory phenotypes [35-37]. The column is filled with specially designed acetate beads as leukocytapheresis carriers [21]. The carriers remove from the blood in the column most of the granulocytes and monocytes/macrophages together with a significant fraction of platelets [15]. In a series of elegant studies, Hanai and colleagues found that patients with active IBD have elevated pro-inflammatory CD14+CD16+DR++ monocytes, which were depleted by granulomonocytapheresis [35]. Further, patients with IBD carry soluble immune complexes (IC) in their plasma. Granulomonocytapheresis carriers adsorb immunoglobulin G (IgG) and IC, and upon adsorption, the binding sites on IgG and IC become available for the fragment crystallizable gamma (Fcγ) receptor (FcγR) on myeloid lineage leukocytes [13,16].

**Table 1:** Peripheral blood neutrophil counts Peripheral blood neutrophil (granulocyte) counts in patients with inflammatory bowel disease (IBD), ulcerative colitis (UC), and Crohn's disease (CD). Proinflammatory cytokines like tumor necrosis factor (TNF)-α, interleukin (IL)-1β, and corticosteroids are known to promote neutrophil survival. Accordingly, certain drugs that are used to treat active IBD potentially may complicate the disease. As seen, Adacolumn granulomonocytapheresis removes up to 60% of granulocytes from the blood, which passes through the Adacolumn. Table adapted from [21] (published under CC BY 4.0 license).

Subjects	Neutrophil count (X10^3^/μL)
Healthy adults	3.4 ± 0.4
Ulcerative colitis	6.4 ± 0.5
Crohn's disease	7.8 ± 1.3
Severe UC	9.3 ± 0.5
Typical granulomonocytapheresis action in severe UC	9.6 pre-granulomonocytapheresis, 4.3 post-granulomonocytapheresis

**Figure 3:**
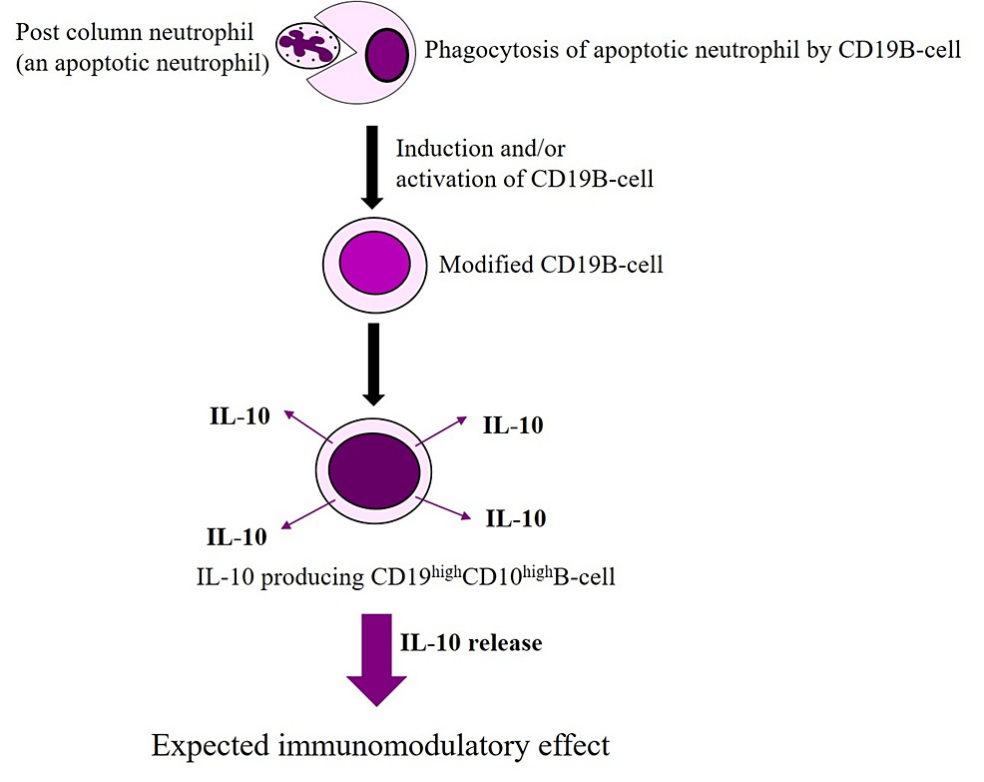
Granulomonocytapheresis-dependent generation of IL-10-producing CD19highCD1Dhigh B-cells (Bregs) A tentative presentation of the Adacolumn granulomonocytapheresis carrier-dependent generation of regulatory CD19^high^CD1D^high^ B-cells (Bregs), experimentally shown by Ansary et al. [35]. Exposure of neutrophils to the granulomonocytapheresis carriers resulted in the generation of apoptotic neutrophils (AN). In clinical settings, a significant fraction of AN (≥40%) re-enters the patients’ circulation via the granulomonocytapheresis column outflow line (blood return to the patient). In the circulation, ANs are phagocytosed by CD19+ B-cells to become interleukin (IL)-10 producing CD19^high^CD1D^high^ B-cells (Bregs). Figure reproduced from Saniabadi et al. [10] J Clin Apher 2019; 34: 51-60 (published under CC BY 4.0 license).

Regarding lymphocytes, an earlier report by Saniabadi et al. showed that granulomonocytapheresis was followed by a sustained increase in absolute lymphocyte counts [21]. Subsequently, Yokoyama et al. found that the increase in lymphocyte count post granulomonocytapheresis therapy of IBD patients included the regulatory CD4(+)CD25(+) phenotype (Treg) [35]. Then, Ansary et al. described granulomonocytapheresis carrier-dependent generation of interleukin (IL)-10-producing regulatory B-cells. Regulatory B-cells (Bregs) are known as CD19^high^CD1D^high^ B-cells [36]. The authors showed that exposure of leukocytes to the granulomonocytapheresis beads resulted in the generation of apoptotic neutrophils [36]. In clinical settings, a significant fraction of apoptotic neutrophils (≥40%) re-enter the patients’ circulation via the granulomonocytapheresis column outflow line that returns to the patient during granulomonocytapheresis [15,21]. In the circulation, the apoptotic neutrophils are phagocytosed by CD19 B-cells, which then become IL-10-producing Bregs, or CD19^high^CD1D^high^ B-cells [36], as shown in Figure 3. IL-10 is a major anti-inflammatory cytokine. Therefore, the remaining fraction of neutrophils that re-enter the circulation via the granulomonocytapheresis column outflow line are potentially the mediators of long-term granulomonocytapheresis efficacy by generating IL-10-producing Bregs.

However, in contrast to elevated myeloid lineage leukocytes seen in patients with active IBD [15,21,34], lymphocytes are very much compromised in IBD patients [15]. This author is not aware of any published data showing elevated circulating lymphocytes in patients with active IBD. Further, in one of the best-controlled studies on lymphocytapheresis in IBD, Lerebours et al. selectively depleted circulating lymphocytes in patients with CD [38]. At the 18 months follow-up, the cumulative relapse rates were 83% in the lymphocytapheresis group, and 62% in the control group, showing a 21% higher relapse rate in the lymphocytapheresis group.

Granulomonocytapheresis in adult patients with IBD

In patients with active IBD, clinical studies in Japan [9,11,14,15,17,39-42], Europe [16,19,20,43-47], and the United States [48,49] have reported on the efficacy and safety of granulomonocytapheresis with Adacolumn (developed by JIMRO, Takasaki City, Japan). Following these evaluation studies, granulomonocytapheresis has been widely applied in clinical practice settings to treat patients with active IBD [16,19,20,43-47]. Efficacy rates reported in real-world clinical practice UC settings have been encouraging [11,14-16], but disappointing findings have also been reported in patients with a long duration of IBD refractory to currently available pharmacological treatments [48,49]. However, low efficacy outcomes were challenged by a more recent retrospective analysis of the same data [45]. Therefore, as mentioned above, an inadequate response to granulomonocytapheresis is thought to reflect patients’ demographic features at baseline, including the severity of mucosal lesions and duration of active IBD after exposure to multiple pharmacological treatments [9,13,15,17]. However, in real-world clinical practice settings, remission rates with granulomonocytapheresis have been as high as 85% in steroid-naïve UC patients [15], and 100% in drug-naïve, first-episode UC cases [50,51]. It is important to state here that early initiation of granulomonocytapheresis before a patient’s IBD has become refractory to multiple pharmacological treatments has been associated with a favorable long-term clinical course [42,52].

Regarding CD, which is the second major phenotype of IBD, experience on the efficacy of granulomonocytapheresis has not been as much as in the UC setting. The first pilot study in CD was reported by Matsui et al., who treated seven patients refractory to conventional medications, of whom five achieved remission [53]. It is appropriate to mention here that the only two non-responders in Matsui et al.'s study had CD confined to the small intestine, which usually does not show extensive myeloid lineage leukocyte infiltration. Subsequently, Fukuda et al. reported a remission rate of 52% in 21 patients with severe CD [54], but a study in patients with CD refractory to currently available conventional medications, including novel biologics, did not show significant efficacy [49]. However, the best efficacy outcome in CD has been reported by Bresci et al. in a cohort of difficult-to-treat patients [20].

Granulomonocytapheresis in pediatrics and adolescents with IBD

Therapeutic outcomes for Adacolumn granulomonocytapheresis in the pediatric IBD populations have been reported by several authors [18,55-58], indicating significant efficacy with a favorable safety profile. Efficacy outcomes with granulomonocytapheresis are stable like medication-induced remission. Indeed, the most recent studies suggest that granulomonocytapheresis holds the potential to bypass the limitations associated with pharmacological treatments [18]. The majority of pediatric and adolescent patients have responded well to granulomonocytapheresis and could avoid pharmacological treatments, without any safety concerns [17,18,57]. The first multicenter study in a small group of patients was undertaken by Tomomasa and colleagues in children with active UC refractory to corticosteroids [55]. In that study, 12 children received one granulomonocytapheresis session per week for five to 10 consecutive weeks. The average UC severity index improved from 2.6 ± 0.3 at baseline to 0.4 ± 0.2 evaluated within two weeks following the last granulomonocytapheresis session. Additionally, the patients’ corticosteroid dose was tapered to a minimum during the time course of granulomonocytapheresis therapy. However, one patient who initially responded subsequently developed bloody diarrhea and two patients remained unchanged. Difficulties were reported in accessing blood vessels for granulomonocytapheresis. In routine settings, the blood flow rate into the granulomonocytapheresis column has been set at 30 mL/min, which can be increased [53,54], but a decrease below 30 mL/min has not been reported. Ikeda et al. applied granulomonocytapheresis to four pediatric/adolescents aged 11 to 17 years with active UC while on conventional drugs, including corticosteroids [56]. Granulomonocytapheresis was introduced with the intention of tapering the corticosteroid dose in these four patients. Patients received one granulomonocytapheresis session per week for five consecutive weeks. Two patients responded well without additional medications, and in another patient, laboratory measures improved and one patient did not respond.

**Figure 4:**
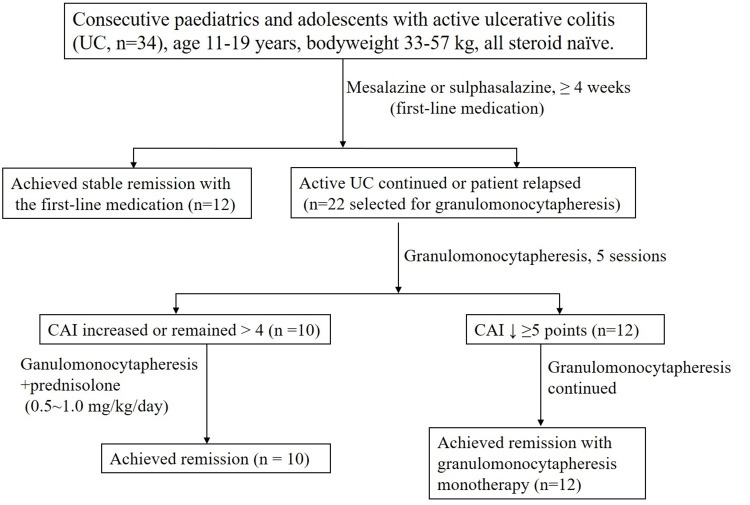
Typical efficacy outcome for the Adacolumn granulomonocytapheresis Typical efficacy outcome for the Adacolumn granulomonocytapheresis at the author’s hospital. A cohort of 34 children initially received mesalazine or sulfasalazine as a first-line medication. Twenty-two patients relapsed or did not respond to the first-line medications, and were selected to receive granulomonocytapheresis, two sessions in the first week and then weekly up to a maximum of 11 sessions. Patients who achieved a decrease of at least five points in the clinical activity index (CAI) score after five granulomonocytapheresis sessions (n = 12) continued receiving additional granulomonocytapheresis sessions, while non-responders to the first five sessions (n = 10) received 0.5 to 1.0 mg/kg body weight/day prednisolone, and continued receiving granulomonocytapheresis. Patients’ ulcerative colitis (UC) activity was evaluated at entry, prior to each granulomonocytapheresis session, and at week 12. Overall, 12 of the 34 patients achieved remission with the first-line medications, another 12 responded to the first five granulomonocytapheresis sessions and continued receiving granulomonocytapheresis without any additional pharmacologic. At entry, the overall CAI score was 14.1 ± 0.4, and the mean endoscopic score was 9.2 ± 0.3. The corresponding values at week 12 were 2.1 ± 0.2 and 2.4 ± 0.2, respectively. Prednisolone dose was tapered to 0 mg within three months in those who did receive this medication. Therefore, at week 12, all 34 patients were in clinical remission, the majority with mucosal healing. Figure created by the author.

Efficacy outcomes in a European retrospective study have been similar to the studies in Japan [58]. Ruuska and colleagues treated 37 children with a mean age of 13 years, 22 of whom had UC, 13 had CD, and two had indeterminate colitis [58]. In that study, the average UC severity index (clinical activity index, CAI) and pediatric Crohn’s disease activity index (CDAI) decreased significantly in both groups after three months, and by the end of the granulomonocytapheresis treatment course, the dose of corticosteroid was significantly reduced in the UC group. The authors concluded that granulomonocytapheresis was an effective and safe treatment in 81% of the steroid-dependent or steroid-refractory pediatric IBD patients [58]. In a subsequent trial (the ADAPT study), Ruuska and colleagues [59] included 25 pediatric and adolescent patients with moderately severe UC. Patients received weekly granulomonocytapheresis sessions over five consecutive weeks, optionally followed by up to three additional sessions over three consecutive weeks (maximum eight sessions). The authors concluded that in pediatric and adolescent patients with moderately active UC, granulomonocytapheresis was effective without safety concerns. Their data supported granulomonocytapheresis as a relevant therapeutic option in pediatric patients who have not responded to first-line drug therapy. In fact, the treatment design at this author’s hospital described in Figure 4 is precisely in line with the statement made by Ruuska et al. that granulomonocytapheresis is a relevant option for patients who have failed first-line medications [58,59].

Granulomonocytapheresis with the Adacolumn as a non-pharmacologic treatment option is generally favored by patients for its safety profile. Reported side effects are usually those often observed during extracorporeal therapy and include transient lightheadedness, mild headache, flushing, nausea, and mild fever in a small fraction of the treated patients [12,17,19,34,41,44,56-62]. In one European study involving a large number of patients [63], the investigators had a major focus on safety, tolerability, and technical feasibility. They reported favorable safety, feasibility, and tolerability for granulomonocytapheresis.

Future prospective

When IBD is viewed from an etiological standing, the condition has features that reflect the presence of elevated or overactive components of the immune system. Therefore, even in this age of modern medicine, the concept of treating disease by removing the causative agents through apheresis is intriguing and can be expanded to minimize the need for drug therapy or potentially as an adjunct to maximize drug efficacy and minimize drug side effects that are most relevant in pediatrics and adolescents. In fact, the efficacy of infliximab has validated the notion that IBD is perpetuated by inflammatory cytokines like tumor necrosis factor-alpha (TNF-α). Others include IL-1β, IL-6, IL-8, and IL-12/23, but major sources of inflammatory cytokines include circulating granulocytes and monocytes/macrophages, which in patients with IBD show activation behavior and prolonged survival time. In particular, depletion of elevated TNF-α producing CD14+CD16+DR++ monocyte phenotype should reduce the TNF-α profile and allow natural healing to proceed, while an increase in the regulatory CD4(+)CD25(+) T-cell and B-cell phenotypes associated with granulomonocytapheresis is potentially very interesting in terms of immunoregulation [64]. The impact of increasing the flow rate (>30 mL/minute) and the duration of one granulomonocytapheresis session (>60 minutes) to achieve a higher processed blood volume needs to be fully evaluated for optimum efficacy outcomes in granulomonocytapheresis therapy.

## Conclusions

Given that IBD is a debilitating chronic disease for which patients require life-long immunosuppressive medication, potentially causing adverse side effects as additional complicating factors, for patients who respond well, granulomonocytapheresis has unparalleled advantages over pharmacological treatments. In fact, clinical experience shows that granulomonocytapheresis has a favorable safety profile, and serious adverse events have been very rare. Further, by selecting patients who are most likely to respond, the futile use of medical resources is avoided.
